# Method for estimating castor seed weight from fruit weight considering the variance of the conversion factor

**DOI:** 10.1016/j.mex.2026.104033

**Published:** 2026-07-10

**Authors:** Liv Soares Severino, Carlos Jorge da Silva, Jose Angelo Nogueira de Menezes

**Affiliations:** a Embrapa Algodão, Sinop, MT, Brazil; b Instituto Federal de Mato Grosso, Campo Novo do Parecis, MT, Brazil; c Embrapa Milho e Sorgo, Sete Lagoas, MG, Brazil

**Keywords:** *Ricinus communis*, Seed yield, Analysis of variance

## Abstract

This method can be employed to estimate the castor seed weight from the castor fruit weight considering that the conversion factor has an intrinsic variance that should be accounted for in the analysis of variance. This method consists in the correction of the Total Sum of Squares when the seed weight is calculated from the fruit weight using a conversion factor and the data is subjected to Analysis of Variance. Conversion factors were measured in 1,000 samples, and the variance of the data was calculated and validated on a diversity of genotypes and experimental conditions.

• Adopting a conversion factor for estimating castor seed weight reduces the labor required for running experiments.
• When castor seed weight is estimated from the fruit weight, the variance of the conversion factor should be accounted for in the Analysis of Variance.
• The conversion factor and its variance may be influenced by genotypes, experimental treatments, and environmental conditions.

## Graphical abstract

**Figure:**
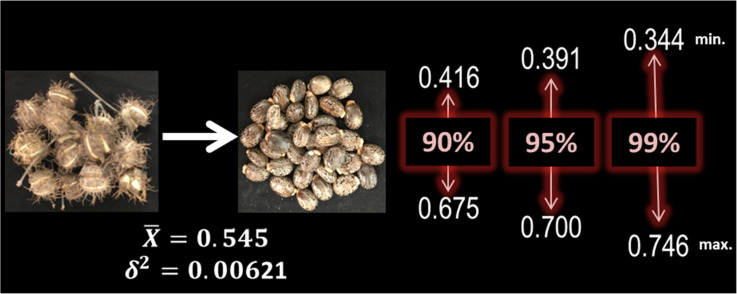

**Specifications table**

**Table:** 

**Subject area**	Agricultural and Biological Sciences
**More specific subject area**	Oilseed crops
**Name of your method**	Variance of the conversion of castor fruit to seed weight
**Name and reference of original method**	Measurement of castor seed weight [1] C. Deepika, S.R. Venkatachalam, A. Yuvaraja, P. Arutchenthil, N. Indra, V. Ravichandran, et. al., Seed morphological characterization, genetic diversity and association analysis in late flowering monoecious lines of castor *Ricinus communis* L, Electronic J. Plant Breed. 13 (2022) 574-583, doi:10.37992/2022.1302.094. [2] J.S. Oswalt, J.M. Rieff, L.S. Severino, D.L. Auld, C.W. Bednarz, G.L. Ritchie, Plant height and seed yield of castor (*Ricinus communis* L.) sprayed with growth retardants and harvest aid chemicals, Industrial Crops Prod. 61 (2014) 272-277, doi:10.1016/j.indcrop.2014.07.006.
**Resource availability**	N/A

## Background information

Castor (*Ricinus communis*) is an oilseed crop that produces seeds containing inedible oil that is largely used in the chemical industry worldwide. Castor oil is a platform chemical used for a long list of products such as lubricants, glues, foam, paints, among many others. As the seed is the commercial product of this crop, the most important variable to be considered in the majority of studies on castor crop is the seed weight, which is also expressed as seed yield or productivity. Castor seed yield is calculated as the seed weight divided by the area in which it was harvested. Productivity is usually expressed using the International System of Units as kilogram per hectare (kg/ha). Measuring the harvested area is easy and precise; however, measuring castor seed weight is labor and time consuming because the samples are harvested as fruit, and the clean seeds need to be removed from the capsules to be weighed. The experiments produce large amounts of fruits to be processed, and the labor required to obtain the clean seed may limit the experimentation with this crop.

This method aims to reduce the labor required for processing the large quantity of castor fruits harvested for measuring seed weight in experiments with castor (*Ricinus communis*). This method consists in a quantitative process to estimate the castor seed weight from the weight of castor fruit samples, but it considers that the conversion factor has a variance that needs to be considered in the statistical procedures.

Weighing samples of castor fruit and using that value to estimate castor seed weight is a method frequently adopted by researchers [[1], [2], [3], [4]], and it is also called in studies of genetic divergence as “shelling percentage”, “shelling out”, or “shelling out turn” [1,5,6]. This method proposes an improvement on that traditional procedure, assuming that the ratio between fruit and seed weights should not be considered a constant, but it is a variable, implying that it varies randomly around a mean with a measurable variance. If the seed yield is calculated multiplying two variables (castor fruit weight multiplied by the conversion factor) the variance of both variables should be accounted for in the statistical analysis. The variance of a product of two independent variables can be calculated by the following approximation [7]:

$$\sigma_{AxB}^{2}={\bar{X}}_{A}^{2}\times \sigma_{B}^{2}+{\bar{X}}_{B}^{2}\times \sigma_{A}^{2}+\sigma_{A}^{2}\times \sigma_{B}^{2},\;\text{where}$$

$\sigma_{AxB}^{2}$ is the variance of the product of *A* x *B*
${\bar{X}}_{A}^{2}$ and ${\bar{X}}_{B}^{2}$ are the squares of the means of the variables *A* and *B*
$\sigma_{A}^{2}$ and $\sigma_{B}^{2}$ are the variance of the of the variables *A* and *B*

## Estimation of the conversion factor and its variance

One thousand samples of castor fruit were obtained from eight experiments with castor plants, performed at field conditions, in the years of 2022 and 2023, in the State of Mato Grosso, Brazil, under a wide range of environmental conditions (locations, genotypes, soil profiles, planting times, and agronomical managements). The experiments were related to doses of fertilizers – N, P, K, and minor nutrients (2 experiments), doses of plant growth regulators – trinexapac-ethyl (1 experiment), evaluation of mechanical harvest efficiency (2 experiments), and variety trials (2 experiments). The results of those experiments are not reported here, but they were just source of castor fruit samples with high diversity. The samples were collected when the fruits were mature (brown color), placed in paper bags, oven dried (65°C for at least 2 days), divided in multiple small fruit samples (in general, samples weighing 50 g), dehulled manually, and the clean seed was weighed. The weight of castor fruits samples varied from 13 to 1,000 g, with an overall average of 53.3 g.

**Fig. 1:**
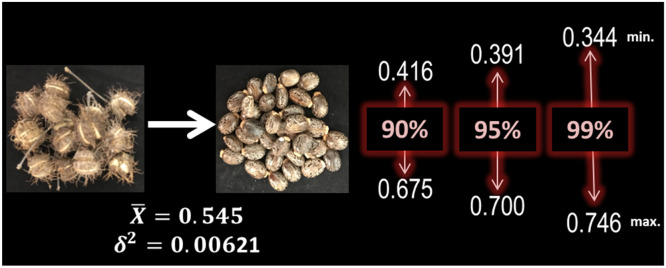
Average and variance of the conversion factor of castor fruit weight to seed weight measured in 1,000 samples, and the intervals of confidence of the conversion factor for the probabilities of 90, 95, and 99%.

Based on the analysis of 1,000 replications, the mean conversion factor of fruit to seed weight was 0.545, and the variance of the conversion factor was 0.00621 (Fig. 1).

**How to estimate the castor seed weight of experimental samples**

1) Each sample of castor fruit harvested in the experiment is weighed individually.
2) Some subsamples of castor fruit are weighed, the fruit husks are removed, and the clean seed is weighed. The conversion factor of each subsample is calculated dividing the clean seed weight by the fruit weight. The values of the conversion factors of the subsamples is used to calculate the mean and the variance of the conversion factor.
3) The castor seed weight of each experimental sample is calculated multiplying its fruit weight by the mean conversion factor.

**How to apply the method in the analysis of variance**

If the castor seed weight is estimated using a conversion factor and the data is subjected to Analysis of Variance, the Total Sum of Squares is calculated as follow:

$$SS_{T}=n\times \;\left({\bar{X}}_{F}^{2}\times \sigma_{C}^{2}+{\bar{X}}_{C}^{2}\times \sigma_{F}^{2}+\sigma_{F}^{2}\times \sigma_{C}^{2}\right),\;\text{where}$$

$SS_{T}\;$is the Total Sum of Squares
n is the number of samples in the experiment
${\bar{X}}_{F}^{2}$ is the square of the mean of the fruit weight
$\sigma_{F}^{2}$ is the variance of the of the fruit weights
${\bar{X}}_{C}^{2}$ is the square of the mean of the conversion factors, and
$\sigma_{C}^{2}$ is the variance of the conversion factors

The method is demonstrated as follow. The example is an experiment in Completely Randomized Design with two treatments (A and B) and 10 replicates (Table 1). The conversion factor was estimated in 16 samples of approximately 80 g of castor fruit (Table 1). The seed weight of each sample was calculated from the fruit weight (Table 2).

**Table 1:** Values of castor fruit weight and castor seed weight on 16 subsamples for the calculation of the conversion factor and its variance.

Subsample	Fruit weight (g)	Seed weight (g)	Conversion factor
1	80.52	45.31	0.56
2	81.35	47.15	0.58
3	82.08	39.74	0.48
4	82.64	51.66	0.63
5	82.54	39.58	0.48
6	82.44	48.28	0.59
7	81.96	43.88	0.54
8	79.75	38.01	0.48
9	81.16	37.31	0.46
10	82.83	41.63	0.50
11	80.55	40.71	0.51
12	82.31	47.14	0.57
13	82.32	39.23	0.48
14	80.91	35.58	0.44
15	78.97	37.45	0.47
16	82.10	41.01	0.50
Mean of the conversion factor:			0.516
Variance of the conversion factor:			0.002708

**Table 2:** Castor fruit and seed weights in an experiment in Completely Randomized Design with two treatments and 10 replicates. The samples of castor fruit were weighed, and the castor seed weight was calculated using the mean conversion factor of 0.516.

Replicate	Treatment A	Treatment B	Treatment A	Treatment B
	Castor fruit weight		Castor seed weight (g)	
R1	190.8	286.5	98.5	147.9
R2	165.1	282.5	85.2	145.8
R3	175.3	218.2	90.5	112.6
R4	199.3	274.5	102.9	141.7
R5	176.1	255.4	90.9	131.8
R6	193.9	299.5	100.1	154.6
R7	174.7	293.2	90.2	151.4
R8	187.2	257.9	96.6	133.1
R9	162.2	267.7	83.7	138.2
R10	166.4	264.1	85.9	136.3
Mean	224.5		92.5	139.4

Analysis of Variance

$${\bar{X}}_{F}^{2}={\left(\frac{190.8+\ldots +264.1}{20}\right)}^{2}=50411.5$$

$$\sigma_{F}^{2}=\left(\sum 190.8^{2}+...+264.1^{2}-\frac{\sum {\left(190.8+\ldots +264.1\right)}^{2}}{20}\right)÷20=\;2386.2$$

$${\bar{X}}_{C}^{2}=={\left(\frac{0.56+\ldots +0.50}{16}\right)}^{2}=0.266$$

$$\sigma_{C}^{2}=\left(\sum 0.56^{2}+...+0.50^{2}-\frac{\sum {\left(0.56+\ldots +0.50\right)}^{2}}{16}\right)÷16=0.002708$$

Total Sum of Squares = 20 x (50411.5 × 0.002708 + 0.266 × 2386.2 + 2386.2 × 0.002708) = 15578.0*

Sum and Mean Squares of Treatments = $\sum \frac{{\left(10\;\times \;92.5\right)}^{2}+{\left(10\;\times \;139.4\right)}^{2}}{10}-\frac{\sum {\left(98.5+\ldots +136.3\right)}^{2}}{20})$ = 10997.7

Sum of Squares of Errors = 15578.0 −10997.7 = 4580.3

Mean Squares of Errors = 4580.3 ÷ (20 – 2) = 254.5

F value = 10997.7 ÷ 254.5 = 43.2

Significance: p < 0.1%

* Just for comparison, if the Total Sum of Squares were calculated with the traditional method of the mean conversion factor, the value would be 12718.0.

**How to apply the method for a single sample**

It is common to harvest and weigh samples of castor fruits and measure a conversion factor to estimate the seed weight or the seed yield of a field. This method proposes estimating an interval of confidence with an associated probability instead of a specific seed weight or yield. Applying this method for that purpose is illustrated as follow. The conversion factor to be used is not the mean (0.545), but the factors on the limits of the interval with 90% of probability: 0.416 and 0.675 (Fig. 1).

A sample of castor fruits was harvested in an area of 1 m^2^ to estimate the castor seed yield of a crop, and it weighed 275.0 g. Then, the seed yield of that field had a probability of 90% to be between 114.4 (275.0 g x 0.416) and 185.6 g/m^2^ (275.0 g x 0.675) (Fig. 1).

If a customized conversion factor is measured to estimate the seed weight of a single fruit sample, it should be measured in several subsamples of fruits in order to estimate its variance. The limits of the interval of confidence can be determined with the traditional statistics of normal distribution.

## Validation of the method with Monte Carlo simulations

This simulation was made to validate the method on extremes of variability of the experimental samples of castor fruit weight and with varying sizes of castor fruit sample. The dataset with 1,000 conversion factors (real values) was employed in a Monte Carlo simulation using artificial data of castor fruit weight. The first simulation was made with 140 artificial datasets of 100 values of castor fruit weight, with$\;\bar{X}=100$, and standard deviation varying in arithmetic progression from 0.25 to 35.00. The second simulation was made with 100 artificial datasets with 100 values of castor fruit weight with mean varying in arithmetic progression from 10 to 1000, and standard deviation of 20% of mean fruit weight. Each artificial value of castor fruit weight was multiplied by one conversion factor randomly assigned from the dataset of 1000 real values. For each dataset, the Total Sum of Squares (SS_T_) was calculated in three ways: (i) according to the method proposed, (ii) considering the mean value of the conversion factor to all the castor fruit samples (easy traditional method), and (iii) considering the seed weight calculated with a different conversion factor assigned to each sample (labor-demanding method).

**Fig. 2:**
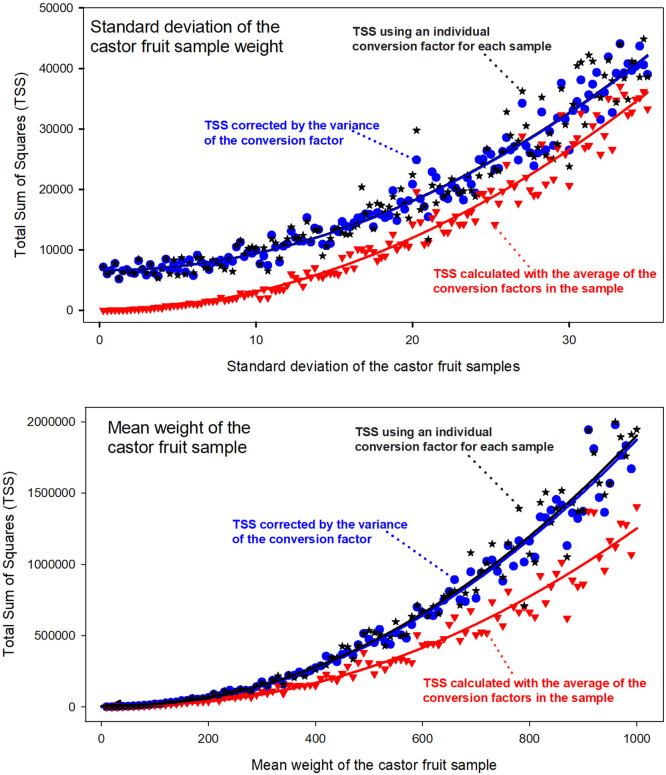
Monte Carlo simulations of the Total Sum of Squares (TSS) obtained from series of castor fruit weight with constant mean $\left(\bar{X}=100\right)$ and standard deviation varying from 0.1 to 35.0 (graph on the top) and mean fruit weight varying from 10 to 1000 (graph on the bottom). The blue dots are the TSS calculated using the method proposed, which considers the variance of the conversion factor. The red triangles are the TSS calculated using the traditional mean conversion factor. The black stars are the TSS calculated using a different conversion factor for each sample of castor fruit.

The Monte Carlo simulations demonstrate that the method proposed returns variances very close to the variances calculated using a different conversion factor for each sample (Fig. 2). The trend lines are overlapping because the values are equivalent. The method was also stable to correct the SS_T_ in samples with wide amplitude of variability and size. The SS_T_ is consistently smaller when castor seed weight is calculated employing a mean conversion factor, although that is the predominant method found in the scientific literature on castor seed yield [[1], [2], [3], [4]].

The method proposed result in SS_T_ very close to the alternative method, in which individual conversion factors are assigned to each sample of castor fruit. It is noteworthy emphasizing that the procedure employed in the option (iii) is not recommended. It is more laborious, and it does not result in higher accuracy than the method proposed.

**Points to consider when using this method**

• Samples of castor fruit must be collected and processed with special attention to details that influence the conversion factor of fruit to seed weight, such as the moisture content of the sample (e.g., oven dried or natural moisture), the presence of green castor fruits, excluding or not aborted (empty) seeds after threshing, and if contaminants and fruit peduncle are sorted out from the fruit sample before weighing. All the sources of variability are reflected in the variance of the conversion factor, and a careful sample preparation favors reducing the variability. The subsamples for measuring the conversion factor and the fruit samples must be in similar conditions regarding all those factors.
• The magnitude and the variance of the conversion factor can be influenced by some experimental treatments, and it is particularly different among castor genotypes. Whenever possible, it must be checked if the conversion factors across treatments are statistically equal (both for its value and variance).
• The quantity and the size of subsamples of castor fruit needed to estimate the conversion factor and its variance is not predetermined. It is a decision of the researcher considering that the accuracy is increased when the conversion factor is measured with more quantity and size of the subsamples.
• The number of subsamples required to estimate the conversion factor is independent of the number of treatments or experimental plots. The objective must be obtaining a reliable estimation of the mean and variance of the conversion factor.
• Measuring a different conversion factor for each experimental plot do not exempt the use of the procedures herein proposed because the conversion factor measured for each plot has an intrinsic variance that must be accounted.
• As alternative, when it is not possible to get a reliable estimation of the mean and variance of the conversion factor, the values presented in this study ($\bar{X}=0.545$ and $\sigma^{2}=0.00621$) may be adopted.

## Ethics statements

The authors declare no ethics restrictions for this study.

## CRediT author statement

**Liv S. Severino**: Conceptualization. Methodology. Data curation. Writing. **Carlos J. Silva**: Methodology. Validation. Reviewing and Editing. **José A.N. Menezes Junior**: Validation. Reviewing and Editing.

## Acknowledgments

To Terasol Oleos Vegetais for financial support (Project SEG 30.20.90.020.00.00).

## Declaration of interests

The authors declare that they have no known competing financial interests or personal relationships that could have appeared to influence the work reported in this paper.

## Data Availability

The raw data used for developing this method is available in the supplementary file.

## Appendix Supplementary materials

Image, application 1
